# No difference in plasticity between different ploidy levels in the Mediterranean herb *Mercurialis annua*

**DOI:** 10.1038/s41598-017-07877-3

**Published:** 2017-08-25

**Authors:** Julia Sánchez Vilas, John R. Pannell

**Affiliations:** 1 Department of Plant Sciences, University of Oxford, Oxford, OX1 3RB UK; 2 Organisms and Environment Division, Sir Martin Evans Building, Cardiff School of Biosciences, Cardiff University, Cardiff, CF10 3AX UK; 3 Department of Ecology and Evolution, Biophore Building, University of Lausanne, CH-1015 Lausanne, Switzerland

## Abstract

Increased phenotypic plasticity for a number of plant traits has been suggested as a possible reason for the success and spread of polyploids. One such trait is a plant’s sex allocation (or gender), which influences its reproductive success directly as a function of the potentially heterogeneous mating prospects in the population. However, it is unknown how polyploidy *per se* might affect plasticity in a plant’s sex allocation. Although there have been numerous comparisons between diploid and (usually) tetraploid taxa, we know very little about how elevated ploidy above the diploid level might affect plasticity. Here, we ask whether different ploidy levels > 2x express different plasticity in the ruderal plant *Mercurialis annua*. We grew tetraploid and hexaploid hermaphrodites under different levels of nutrient availability and compared their reaction norms for growth (above-ground biomass, SLA) and reproductive traits (reproductive effort, phenotypic gender). Overall, we found that an increase in ploidy level from 4x to 6x in *M*. *annua* is associated with an increase in the relative biomass allocated to seeds, measured as female reproductive effort. However, our study provides no support for the idea that increasing ploidy level increases the ability to express different phenotypes in response to changes in the environment.

## Introduction

Polyploidy is common in angiosperms, in which most lineages reflect one or more whole genome duplication events^1, 2^. These genome duplication events – particularly allopolyploidy (the combination of two or more distinct genomes through hybridization of two different species) – are widely recognized as a key contributor to the success of angiosperms, the largest clade of land plants^2^. Both allopolyploids and autopolyploids (species with a duplicated whole genome) often enjoy wider geographical ranges and/or occupy distinct habitats compared with their diploid ancestors^3–9^. There are a number of modifications associated with genome duplication, including larger cells and greater plant sizes, which can ultimately result in distinct physiology and ecology^10^. A major likely advantage of polyploidy *per se* arises from the possibility that duplicated gene copies can evolve to assume new or slightly varied functions^11, 12^. Different expression of homologous genes has been found not only for allopolyploids^12^, but it has also been suggested for autotetraploids with enhanced tolerance to salinity^13^. In addition, both allopolyploids and autopolyploids have increased heterozygosity, which in turn may be associated with increased plant vigour (or heterosis)^14–16^ and increased biochemical flexibility^17, 18^. Increased genome flexibility, plant vigour and biochemical flexibility may allow polyploids to enjoy broader ecological tolerance and occupy a wider range of environments^10, 19–22^, helping to explain their evolutionary success^23, 24^. In other words, their putative broader ecological tolerance may be due to their greater ability to express different phenotypes in response to changes in the environment, i.e., phenotypic plasticity^10^.

Phenotypic plasticity can act in different ways. On the one hand, it can confer upon genotypes a high fitness that remains relatively constant across a broad range of environments, including poor environments; this is also known as the ‘Jack-of-all trades’ strategy^25^. Strictly, such genotypes could be considered to be non-plastic for fitness; however, at an individual level such homeostasis must reflect some flexibility for other components of the life history or physiology (e.g., physiological plasticity)^26^. On the other hand, plasticity can allow plants to respond to favourable conditions, expressing different phenotypes in different environments; this ‘Master-of-some’ strategy may give a competitive advantage to plastic genotypes in new environments^25^. An ideal ‘general-purpose genotype’ (sensu)^27^ would be a combination of both situations, showing a non-plastic fitness response to unfavourable conditions, but sufficient plasticity to take advantage of favourable environments^25^.

Increased phenotypic plasticity has been suggested as a mechanism to explain the success and spread of polyploids^10^, but this hypothesis has rarely been empirically tested. Moreover, those studies explicitly comparing plasticity among different ploidy levels rarely provide support for this hypothesis (e.g., refs 28–31, but see ref. 32). The presence of individuals with different ploidy levels within the same species and sexual system allows us to discern the role that phenotypic plasticity plays in the success of polyploids without the cofounding effects of phylogeny and mating context^33^. In addition, most studies focus on comparisons between polyploids and diploids^28–30^, but it has rarely been questioned what the advantages of increasing ploidy levels from, say, tetraploids to hexaploids, might be (but see refs 34–36). This is an important gap, because polyploid complexes often involve a range of ploidy levels (e.g., refs 35, 37 and 38).

Here, we take advantage of the variation in ploidy levels within hermaphrodite populations of the annual herb *Mercurialis annua* to assess the effect of differences in ploidy level on the expression of plasticity in growth and sex allocation of hermaphrodite individuals. Plasticity in sex allocation, i.e., plasticity in the resources allocated to male versus female reproductive functions, may benefit plants both in terms of advantages of the Jack-of-all-trades and the Master-of-some strategies. It can also influence the evolution of dimorphic sexual systems in plants, by affecting both the likelihood that unisexual individuals establish in hermaphroditic populations, as well as the maintenance of hermaphrodites when unisexuals are abundant^39, 40^. Indeed, plasticity in sex allocation is common in hermaphroditic plants, often associated with variation in mate availability^41, 42^, plant size and/or resource status^43–48^. Because polyploids differ not only in size^49^, but also in patterns of allocation of biomass (e.g., to roots)^50^ compared to diploids, we might also expect to observe differences in allocation to reproduction with ploidy level. However, the extent to which polyploidy might play a role in increasing phenotypic plasticity in sex allocation in poorly known, yet such knowledge would contribute to an understanding of the basis of associations between ploidy and gender (e.g., gender dimorphism appears to be more common among polyploid than diploid lineages)^51^.

*M*. *annua* offers ideal material for addressing the issues identified above. First, although hexaploid populations are thought to be the result of allopolyploid hybridization between autotetraploid *M*. *annua* and a diploid relative *M*. *huetii*, all three taxa (tetraploid and hexaploid *M*. *annua*, and diploid *M*. *huetii*) are very closely related and occupy very similar habitats, with overlapping geographic distributions. Tetraploids and hexaploids of *M*. *annua* thus have two versus three copies of a very similar genome, respectively, and we speculate that the additional copy of an extra similar genome contributes as much or more to any differences between the two species than the difference between the genomes involved. Second, sex allocation in polyploid *M*. *annua* is known to be plastic and responsive in its expression to density^52^, nutrients^33, 53^ and light^54^. It is also easy to measure (see Methods). Because *M*. *annua* is wind-pollinated, the relative production of pollen probably relates quite closely to realised reproductive success through siring success, so that plastic responses in sex allocation are likely to have been under strong selection.

Our study addressed the following questions: (i) Do polyploids differing in the number of their chromosome sets (tetraploids and hexaploids) express plasticity differently? Given that hexaploids have an extra set of chromosomes, we may expect that they exhibit greater plasticity in their response to the environment. If so, (ii) do higher ploidy levels (6x) show more of a Jack-of-all trades or a Master-of-some strategies than lower ploidy levels (4x)? If we assume that an extra set of chromosomes confers greater flexibility, we may expect higher ploidy levels to express more of a ‘general purpose genotype’, i.e., a combination of both Jack-of-all trades and Master-of-some strategies, showing higher and non-plastic trait values in response to unfavourable conditions but showing an increase in trait values (plasticity) under favourable environments. We sampled hermaphrodites from several tetraploid and hexaploid populations of *M*. *annua* and grew them in a common environment under different levels of nutrient availability. We assessed their reaction norms (i.e., phenotypic response to different environmental conditions), both in terms of absolute measures of allocation, including total and above-ground vegetative biomass, male, female and total reproductive efforts (MRE, FRE and TRE, respectively) and specific leaf area (SLA, leaf area per unit leaf dry mass), as well as in terms of their relative allocation to male versus female functions, i.e., in terms of their phenotypic gender (*PG*).

## Results

### Biomass and specific leaf area

**Figure 1:**
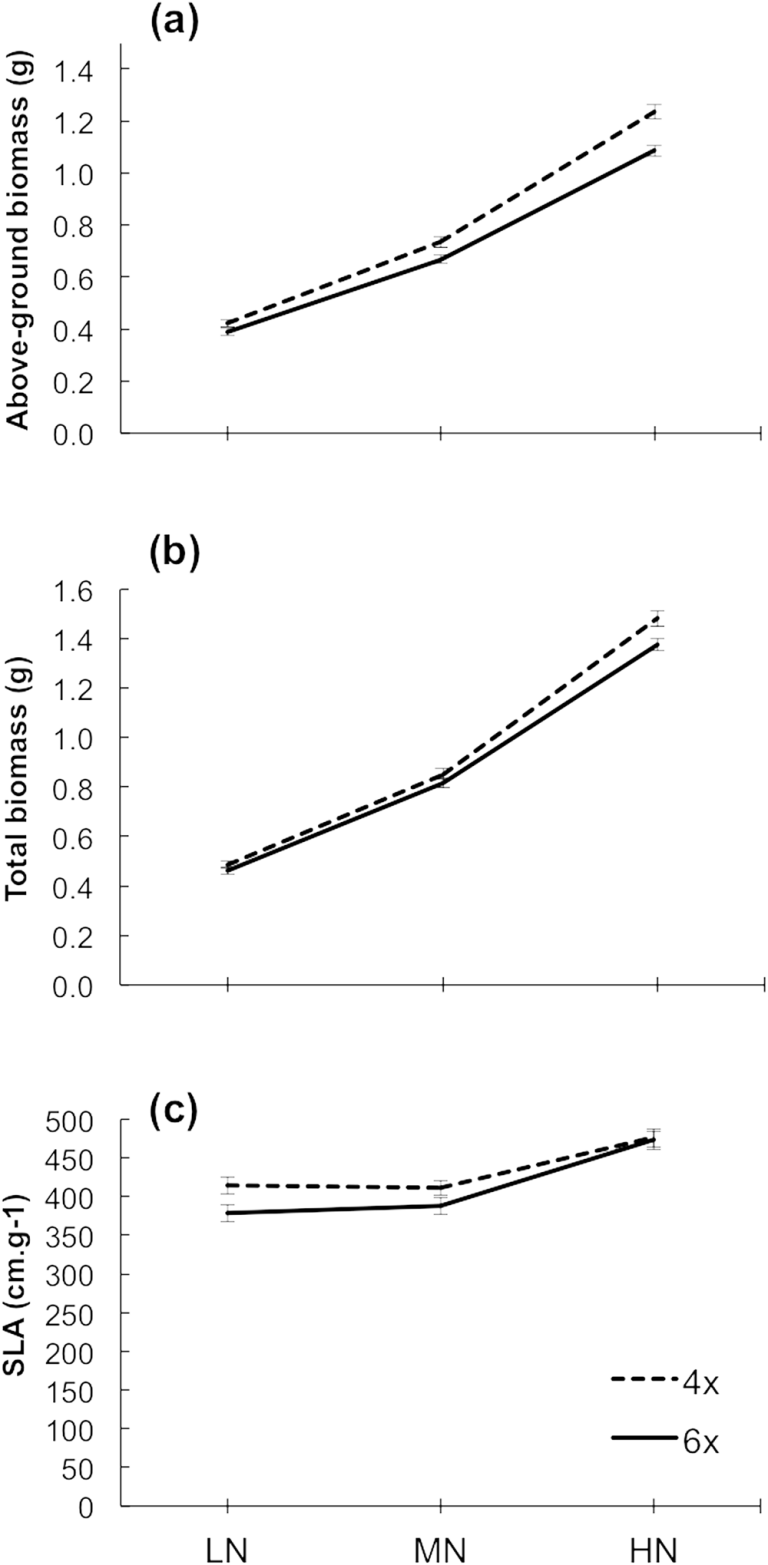
(**a**) Above-ground, (**b**) total biomass, and (**c**) specific leaf area, SLA, at three different levels of nutrient addition (0, 0.3 and 0.9 g L^−1^) for tetraploid and hexaploid hermaphrodites of *M*. *annua* (dashed and solid lines, respectively). For above-ground biomass, the values shown are back-transformed means of log transformed data; raw means are shown for total biomass and specific leaf area.

Levels of moderate and high nutrient availability significantly increased the above-ground and total biomass of the plants (Table 1, Fig. 1a,b). Tetraploids accumulated more above-ground biomass than hexaploids, regardless of resource availability (Table 1, Fig. 1a). However, no differences were found in total biomass for ploidy level, although tetraploids tended to have higher total biomass than hexaploids at higher levels of nutrient availability (Nutrients × ploidy, Table 1, Fig. 1b). High nutrients also increased the specific leaf area of the plants, but no differences were detected between ploidy levels (SLA, Table 1, Fig. 1c).

**Table 1:** Results of linear mixed effects models for above-ground and total biomass (g), and specific leaf area (SLA, cm^2^.g^−1^).

Source	Sum of Squares	df (num, den)	F-value	P-value
Above-ground biomass				
Initial height	0.150	1, 317	19.4	**<0**.**001**
Nutrients	12.7	1, 337	1640	**<0**.**001**
Ploidy	0.0475	1, 8	6.12	**0**.**038**
Nutrients × ploidy	0.002	1, 335	0.256	0.613
Total biomass				
Initial height	0.504	1, 345	19	**<0**.**001**
Nutrients	54.2	1, 337	2007	**<0**.**001**
Ploidy	0.053	1, 8	1.96	0.199
Nutrients × ploidy	0.100	1, 335	3.74	0.054
SLA				
Nutrients	364839	1, 336	53.7	**<0**.**001**
Ploidy	9963	1, 8	1.47	0.260
Nutrients × ploidy	16416	1, 335	2.43	0.120

Degrees of freedom (Satterthwaite approximation), type III SS and P-values were calculated using *lmerTest* ^73^. *P*-values for main factors were obtained after removing non-significant interactions from the model.

### Reproductive effort

**Figure 2:**
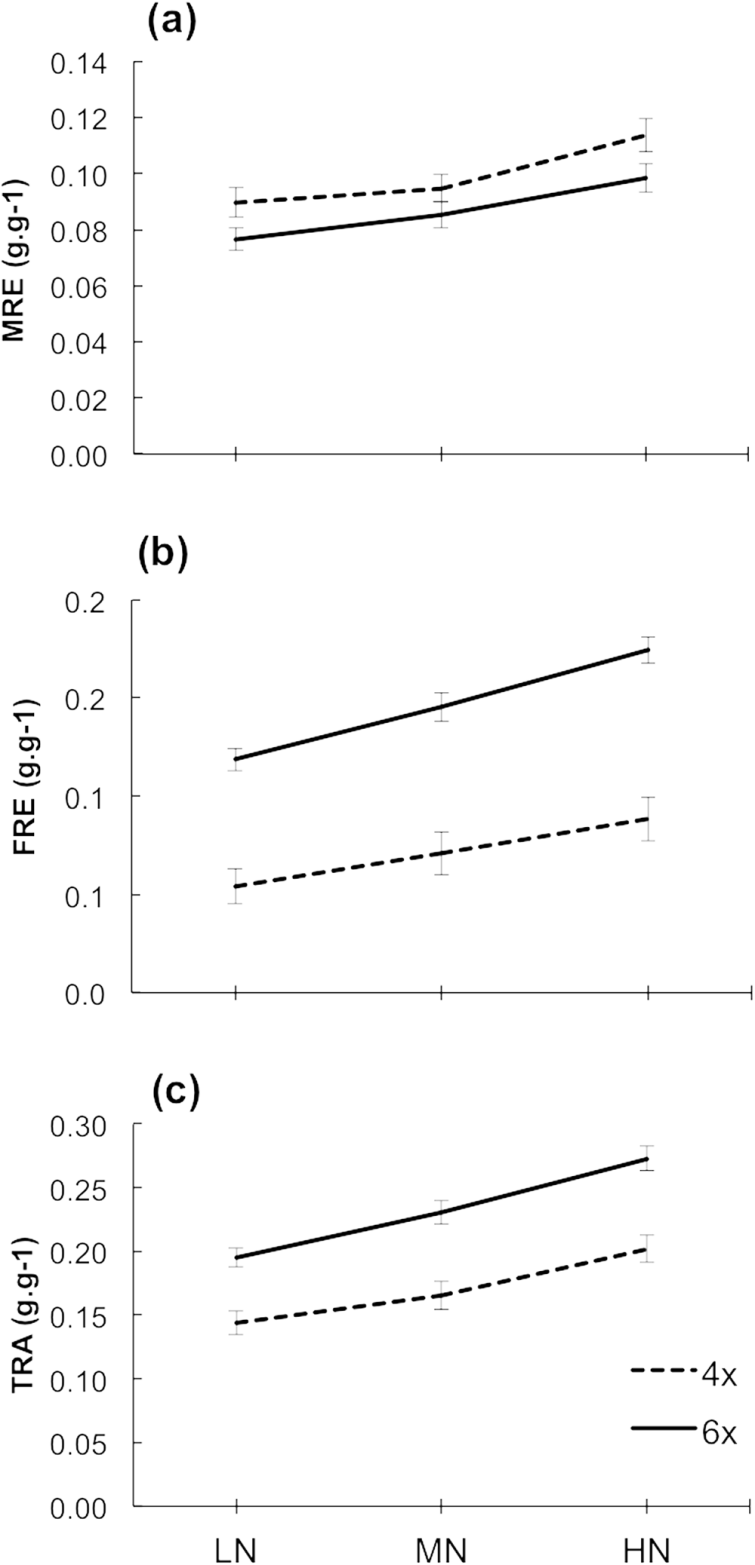
(**a**) Male, (**b**) female and (**c**) total reproductive effort at three different levels of nutrient addition (0, 0.3 and 0.9 g L^−1^) for tetraploid and hexaploid hermaphrodites of *M*. *annua* (dashed and solid lines, respectively). Values shown for FRE are raw means; for MRE and TRE back-transformed means of log transformed data are shown.

Increasing nutrient availability increased allocation to reproduction – to both male and female functions (Table 2, Fig. 2). Hexaploid individuals allocated more biomass to reproduction than tetraploids (Table 2, Fig. 2). This is mainly the result of hexaploids allocating more biomass to their female function (Table 2, Fig. 2b), as no significant differences in allocation of biomass to male function were found between tetraploid and hexaploid individuals (Table 2, Fig. 2a). There was no interaction between treatment and ploidy (Table 2, Fig. 2).

**Table 2:** Results of linear mixed effect models for the male, female and total reproductive effort (MRE, FRE and TRE, respectively) and phenotypic gender (*PG*).

Source	Sum of Squares	df (num, den)	F-value	P-value
MRE				
Nutrients	0.701	1, 348	22.1	**<0**.**001**
Ploidy	0.135	1, 8	4.27	0.073
Nutrients × ploidy	0.0008	1, 347	0.0249	0.875
FRE				
Nutrients	0.123	1, 337	34.4	**<0**.**001**
Ploidy	0.0529	1, 8	14.8	**0**.**005**
Nutrients × ploidy	0.0068	1, 336	1.91	0.168
TRE				
Nutrients	1.47	1, 337	61	**<0**.**001**
Ploidy	0.288	1, 8	11.9	**0**.**009**
Nutrients × ploidy	1.44 × 10^−5^	1, 336	0.0001	0.994
*PG*				
Above-ground biomass	0.116	1,351	3.92	**0**.**048**
Nutrients	0.241	1,349	8.15	**0**.**005**
Ploidy	0.345	1,8	11.6	**0**.**009**
Nutrients × ploidy	0.0269	1,336	0.906	0.342

Degrees of freedom (Satterthwaite approximation), type III SS and P-values were calculated using *lmerTest* ^73^. *P*-values for main factors were obtained after removing the non-significant interactions from the model.

### Experiment-wide phenotypic gender

**Figure 3:**
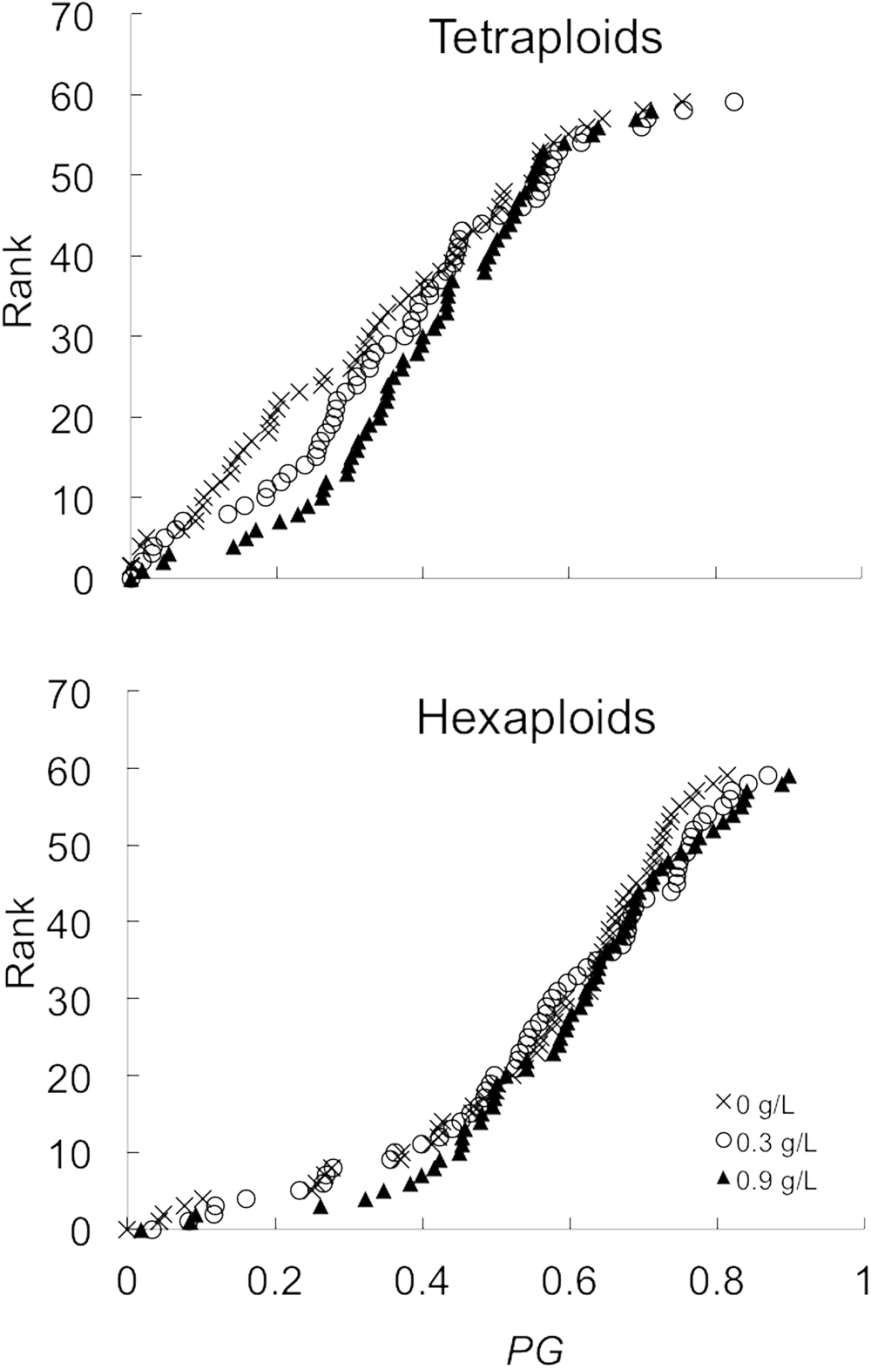
The distribution of phenotypic gender in tetraploid and hexaploid hermaphrodites of *M*. *annua* growing under three levels of nutrient addition (0, 0.3 and 0.9 g L^−1^). The standardized phenotypic femaleness (*PG*) is graphed against its individual rank. N = 60 for all curves, except for tetraploids growing at 0.3 g L^−1^ where N = 59. Plants with a *PG* value of 0 and 1 are strictly male and female, respectively. See text for further details.

There was a significant effect of nutrient availability in terms of experiment-wide phenotypic gender (*PG*, Table 2), with hermaphrodites growing at higher nutrient availability being more female than those growing at lower nutrient availability (Fig. 3). There were also significant differences between ploidy levels in *PG* (Table 2), with tetraploids being more male than hexaploids (Fig. 3). However, there was no significant interaction between nutrients and ploidy (Table 2).

### Coefficient of variation and phenotypic plasticity index

Overall, there were no significant differences between tetraploids and hexaploids in their mean coefficient of variation (CV; t-test = 0.419, P = 0.689) or phenotypic plasticity index (PPI; t-test = 0.149, P = 0.884). According to both the CV and PPI, the most plastic variables were above-ground biomass, total biomass, and FRE (Table 3). *PG* was low for PPI, whereas it was high for CV (Table 3). For most variables, the tetraploids had higher CV and PPI than the hexaploids (Table 3).

**Table 3:** Coefficient of variation (CV) and phenotypic plasticity index (PPI), (maximum - minimum)/maximum, for tetraploids (4×) and hexaploids (6×) for above-ground biomass and total biomass (g), specific leaf area (SLA, cm^2^.g^−1^), male, female and total reproductive effort (MRE, FRE and TRE, respectively) and phenotypic gender (*PG*).

	CV		PPI	
	4×	6×	4×	6×
Above-ground biomass	46.64	44.38	0.655	0.635
Total biomass	47.8	46	0.671	0.658
SLA	21.51	23.12	0.137	0.200
MRE	5.31	5.12	0.211	0.222
FRE	74.04	56.77	0.387	0.321
TRE	8.95	12.23	0.288	0.285
*PG*	53.41	35.52	0.174	0.070
Mean (±S.E.)	36.81 ± 9.61	31.88 ± 7.18	0.359 ± 0.084	0.342 ± 0.084

The average mean value (±S.E.) for each ploidy level is shown in the last row.

## Discussion

Hermaphrodite individuals of *M*. *annua* responded plastically to changes in nutrient availability by increasing their allocation to biomass and reproduction with increasing nutrient availability (i.e., showing a Master-of-some strategy). Overall, large plants were also more male, as found previously for *M*. *annua* ^33^. Nutrient availability also influenced phenotypic gender (*PG*) even when the effect of plant size on *PG* was accounted for, with a shift towards increased femaleness with increasing nutrient availability. This result suggests that, at equal plant size, plants allocated more resources to female function when resources were more available.

We found no evidence of significant differences in plasticity between tetraploid and hexaploid individuals of *M*. *annua* (i.e., there was no significant ploidy × environment interaction, nor were there significant differences in mean CV and PPI). Our study, therefore, provides no support for the idea that increasing chromosome number, *per se*, confers increased genome flexibility. Accordingly, we found no evidence that genome duplication may promote the evolution of gender dimorphism via increased sex allocation plasticity^55^. Most previous studies comparing plasticity of diploids and polyploids have also failed to detect differences^28–31^. These studies have focused on autopolyploids, and the only empirical support for increased phenotypic plasticity associated with polyploids is provided by one study of the allopolyploid *Centaurea stoebe* ^32^.

The origin of the subgenomes of a polyploid lineage, i.e., near-identical in autopolyploids *vs*. more divergent in allopolyploids, may play a potentially important role in creating greater genomic change and variation and hence greater flexibility to cope with a broader array of environmental conditions^56^. However, our study of hexaploids with an allopolyploid origin in *M*. *annua* does not support a link between hybrid-related effects and phenotypic plasticity. As suggested in the Introduction, this may be because hexaploids of *M*. *annua* are composed of very similar chromosome sets to the tetraploids. In the *M*. *annua* complex, the different ploidy levels (diploids, tetraploids, and hexaploids) differ in their geographical distribution, and diploids and hexaploids are ecologically differentiated^57^. Here, our results also point to some degree of ecological differentiation between tetraploids and hexaploids (see discussion below). The tetraploid and hexaploid populations chosen in our study occurred in close proximity, and it is therefore likely that populations from both ploidy levels have experienced similar environmental selective pressures in the recent past. In particular, it is likely that populations from both ploidy levels have been exposed to similar fluctuations in the level of nutrients, and the lack of differences between them in terms of plasticity may thus reflect convergent evolution in a phenotypic response to similar environments^58, 59^.

Regardless of resource availability, tetraploids had greater or similar trait values than hexaploids for all traits measured, except for female and total reproductive effort. Interestingly, diploids of *M*. *annua* have also been found to be superior to hexaploids in several important physiological and life-history traits^60^, which, together with our results, suggest that increasing ploidy level in this species may not confer ‘advantages’ in comparison with lower ploidy levels. However, the hexaploids allocated relatively more biomass to seeds than tetraploids, despite the fact that the two ploidy levels did not differ in above-ground biomass. Assuming low genomic divergence between the genomes involved, the greater seed production of hexaploids may be a direct consequence of genome duplication *per se* (e.g., via an increase in organ size caused by an increase in nuclear DNA content)^61, 62^. We cannot exclude the possibility that differences in mean traits values have evolved in response to geographical or ecological differences, but populations for both ploidy levels chosen in this study occur in close proximity, as noted above. Additionally, the greater femaleness observed in hexaploids may reflect their different recent evolutionary history compared with that of tetraploids. In *M*. *annua*, tetraploids are only found in monoecious populations, but hexaploids can be found in both monoecious and androdioecious populations, where hermaphrodites may co-occur with males^63^. Differences in their past mating environment, i.e., hexaploid hermaphrodites that have been evolving in the presence of males, may have shifted their sex allocation towards greater femaleness than those (tetraploids) that have been evolving in the absence of males (see refs 33 and 64).

## Methods

### Species

*Mercurialis annua* L. (Euphorbiaceae) is a wind-pollinated annual-herb that occupies disturbed habitat all over northern Europe and around the Mediterranean Basin^65, 66^. *M*. *annua* displays a remarkably broad variation in its sexual system including dioecious, monoecious and androdioecious populations along its range of distribution^60, 65^. In addition to this variation in sexual system, populations of *M*. *annua* differ also in their ploidy levels^60, 63, 65, 67^. Fully dioecious populations are exclusively diploid, and these are widespread throughout Europe. In contrast, populations containing hermaphrodites (with or without males) are polyploids and are largely restricted to the western Mediterranean Basin and northwest Africa. Tetraploids of *M*. *annua* occur south of Rabat on the Atlantic coast of Morocco, and these meet with hexaploid populations to the north. Tetraploids are hermaphroditic and of autopolyploid origin, whereas the hexaploids, which are very widespread in northwestern Morocco and around the coast of the Iberian Peninsula, are variously hermaphroditic or androdioecious and of allopolyploid origin^63^. Hexaploid populations of *M*. *annua* meet their diploid counterparts at two contact zones in northeastern and northwestern Spain^63, 65^.

### Experimental design

Plant material was obtained from seeds collected from 5 tetraploid (F17 S Cap Beddouza 32.5450, −9.2694; F20 N El Oualidia 32.7072, −9.0681; F29 N Tnine-des-Chtouka 33.3826, −8.2173; F30 N Tnine-des-Chtouka 33.3996, −8.1719; F31 Tamaris Plage 33.5282, −7.8142) and 5 hexaploid (F35 Oued Mellah 33.5900, −7.6025; F36 N Gueimame 33.7514, −7.2265; F37 N Bouznika 33.7933, −7.1608; F38 N Bouznika 33.8140, −7.1121; F39 North Skirat 33.8745, −6.9953) monoecious populations at North of Morocco, whose ploidy level was previously determined^53^. At each population, seeds were bulk-collected from approx. 40–50 hermaphrodites widely spaced. Seeds were randomly sown in seed trays and grown in glasshouse conditions in the Department of Plant Sciences (University of Oxford). Two weeks after germination, 360 seedlings were transplanted into 10 × 10 × 9 cm pots containing nutrient-poor sandy soil (Silvaperl Sharp Sand, William Sinclair Horticulture, Lincoln, UK). Initial height was recorded and 36 plants per population were randomly assigned to different experimental treatments (12 replicates to each): low, moderate and high nutrient concentrations. Nutrient concentrations were chosen as representative of the range of habitats that *M*. *annua* occupies (from very poor, as found in walls, to nutrient-rich, in cultivated ground)^65, 66^. Plants growing under the low-, medium- and high-nutrient treatments were watered once a week with 75 ml of a solution of 0.0, 0.3, and 0.9 g/L, respectively, of Phostrogen Fertilizer (14:10:27 NPK, Bayer CropScience Limited, Cambridge, UK). Additional water was supplied between nutrient applications, once a week. Saucers were placed under the pots in order to avoid treatment interference. After 5 weeks of growing under experimental conditions, we recorded the final height and harvested the above-ground portions of the plants. Harvested plants were separated into vegetative parts (stems and leaves), and male and female reproductive structures (male flowers, and female flowers and fruits, respectively). Note that the seed set in hermaphrodites of *M*. *annua* is high (approx. 70% of flowers set seed)^68^. One leaf per plant (chosen from the last pair of fully expanded leaves) was scanned and its area determined using image analysis software (ImageJ 1.42q)^69^. The biomass of vegetative and reproductive structures was recorded after oven-drying at 70 °C for 5 days. Male, female and total reproductive efforts (MRE, FRE and TRE, respectively) were calculated by dividing the biomass of the male, female and total reproductive structures by the above-ground vegetative biomass. Specific leaf area was calculated as leaf area divided by leaf dry mass (SLA, cm^2^.g^−1^).

To assess differences in sex allocation among treatments, we calculated a measure of gender for each individual standardized against the average gender of plants across the whole experiment. This experiment-wide phenotypic gender (*PG*), was calculated using the formula proposed by^70, 71^, i.e., *PG*_*i*_ = *d*_*i*_/(*d*_*i*_ + *l*_*i*_ *E*), where *d*_*i*_ is the maternal allocation of individual *i* and *l*_*i*_ is the paternal allocation of individual *i*, and *E* = Σ *d*_*i*_/Σ *l*_*i*_ is the ratio of maternal to paternal allocation summed over plants across the entire experiment. Note that Lloyd’s^70^ measure of phenotypic gender for individuals sampled from a population depends on the frequency distribution of the sex allocation of all other individuals in the same population and is always centered around 0.5. In contrast, our approach centers *PG* around 0.5 for the whole experiment but allows *PG* averaged across individuals within a treatment to deviate from 0.5, allowing comparisons in *PG* among treatments. Maternal and paternal allocations were measured as the dry weight of female and male reproductive structures, respectively.

### Data analysis

We tested for differences between ploidy levels (tetraploid, hexaploid) in response to nutrient availability (0, 0.3 g/L and 0.9 g/L) for male reproductive effort (MRE, male reproductive biomass divided by above-ground biomass), female reproductive effort (FRE, female reproductive biomass divided by above-ground biomass), total reproductive effort (TRE, total reproductive biomass divided by above-ground biomass), phenotypic gender (*PG*), above-ground vegetative biomass, total biomass (above-ground vegetative biomass and reproductive biomass) and specific leaf area (SLA, leaf area per unit leaf dry mass, cm^2^.g^−1^). Ploidy level was fitted as a fixed factor, and nutrient availability was fitted as a continuous covariate. Initial height at the time of randomization was also included as a covariate but removed when non-significant. To analyse the effect of size on *PG*, above-ground biomass was included in the model as covariate. Block and population nested within ploidy level were fitted as random effects. Population was nested within ploidy level because each population can only have one value for ploidy. All analyses were carried out using linear mixed-effects models in the statistical package R using the lmer function^72^. Degrees of freedom (Satterthwaite approximation), type III SS and P-values were calculated using *lmerTest* ^73^. MRE, TRE and above-ground biomass were log_10_-transformed to achieve Normality of standardized residuals and homogeneity of variance. Differences in plasticity between ploidy levels were evaluated by means of the interaction ploidy level × nutrient availability. In addition, a coefficient of variation (CV) and an index of phenotypic plasticity (PPI) were calculated for each variable and ploidy level. PPI ranged from zero to one, and was calculated as the difference between the maximum and minimum mean values across the three nutrient levels and the maximum mean value^74^. Student’s t-tests at the P < 0.05 level were used to determine the effect of ploidy in CV and PPI. All statistical analysis were carried out in R v. 2.8.1^72^.
